# Role of MicroRNAs in Controlling Gene Expression in Different Segments of the Human Epididymis

**DOI:** 10.1371/journal.pone.0034996

**Published:** 2012-04-12

**Authors:** Clémence Belleannée, Ezéquiel Calvo, Véronique Thimon, Daniel G. Cyr, Christine Légaré, Louis Garneau, Robert Sullivan

**Affiliations:** 1 Centre de Recherche du CHUQ and Département d'Obstétrique-Gynécologie, Faculté de Médecine, Université Laval, Québec, Canada; 2 Laboratory of Endocrinology and Genomics, CHUL Research Center and Department of Molecular Medicine, Université Laval, Québec, Canada; 3 Département de Biologie, Université de la Martinique, Martinique, France; 4 INRS-Institut Armand-Frappier, Université du Québec, Laval, Québec, Canada; Clermont-Ferrand Univ., France

## Abstract

**Background:**

The molecular mechanisms implicated in regionalized gene expression in the human epididymis have not yet been fully elucidated. Interestingly, more than 200 microRNAs (miRNAs) have been identified in the human epididymis and could be involved in the regulation of mRNA stability and post-transcriptional expression in this organ.

**Methods:**

Using a miRNA microarray approach, we investigated the correlation between miRNA signatures and gene expression profiles found in three distinct regions (caput, corpus and cauda) of human epididymides from 3 donors. *In silico* prediction of transcript miRNA targets was performed using TargetScan and Miranda software's. FHCE1 immortalized epididymal cell lines were cotransfected with mimic microRNAs and plasmid constructs containing the 3′UTR of predicted target genes downstream of the luciferase gene.

**Results:**

We identified 35 miRNAs differentially expressed in the distinct segments of the epididymis (fold change ≥2, P-value≤0.01). Among these miRNAs, miR-890, miR-892a, miR-892b, miR-891a, miR-891b belonging to the same epididymis-enriched cluster located on the X chromosome, are significantly more expressed in the corpus and cauda regions than in the caput. Interestingly, a strong negative correlation (*r* = −0,89, P-value≤0.001) was found between the pattern of expression of miR-892b and its potential mRNA target Esrrg (Estrogen Related Receptor Gamma) and with miR-145 and Cldn10 mRNA (*r* = −0,92, P-value≤0.001). We confirmed that miR-145 and miR-892b inhibit the expression of the luciferase reporter *via* Cldn10 and Esrrg 3′ UTRs, respectively.

**Conclusion:**

Our study shows that the expression of miRNAs is segmented along the human epididymis and correlates with the pattern of target gene expression in different regions. Therefore, epididymal miRNAs may be in control of the maintenance of gene expression profile in the epididymis, which dictates segment-specific secretion of proteins and establishes physiological compartments that directly or indirectly affect sperm maturation and fertility.

## Introduction

Immature testicular spermatozoa acquire their motility and fertilizing ability during their transit through the epididymis. In humans, this organ is a 6-meter long convoluted tubule that connects the testis to the vas deferens and is composed of three main anatomical regions: the caput, corpus and cauda. Once released from the testis, spermatozoa are concentrated by a mechanism of fluid resorption in the efferent ducts/proximal caput, acquire their motility and fertilizing ability transiently in the caput and corpus regions, and reach within one week the cauda region where they are stored and kept in quiescent state before ejaculation. Each of these regions possesses distinctive gene expression profiles [1]–[5] that dictate segment-specific secretion of proteins into the luminal fluid [6]–[14] and establish physiological compartments that directly or indirectly affect sperm maturation [15]–[18]. The molecular mechanisms resulting in the establishment of regionalized gene expression in the epididymis involve numerous and complex regulatory elements that have not yet been fully elucidated [19], [20]. Among the more important regulatory factors studied, androgens regulate epididymal gene expression either via their interaction with the androgen receptor, which regulates transcription of androgen-dependent genes [21], or via androgen-dependent intracellular signaling pathways [22]. Other factors, such as estradiol, and testicular derived “lumicrine factors” have also been shown to regulate epididymal gene expression and affect primarily the proximal regions of the epididymis [23], [24]. However, the role of RNA interference and the regulation of mRNA stability and gene expression in the epididymis is not well understood.

microRNAs (miRNAs) are small non-coding RNAs (19–22 nucleotides) that modulate gene expression at the post-transcriptional level. These micromolecules bind to target mRNAs at specific sequence motifs within the 3′ untranslated region (3′UTR) of the transcripts. This process is typically mediated by the miRNA seed region, between the second and eighth nucleotides from the 5′ end of a miRNA sequence [25]–[31]. Depending upon the physiological status of the cell, the mRNA/miRNA duplex can either (1) inhibit translation by affecting the initiation step, (2) increase degradation of the target mRNA, or (3) increase translation and protein synthesis [32]–[35]. The mechanism by which miRNAs regulate gene expression is complex, since one miRNA can target several thousand mRNAs, and one mRNA can be regulated by several miRNAs [26]. The fact that miRNA target motifs are highly conserved among species and can regulate more than 30% of all human genes highlights the importance of these small molecules in biological systems.

miRNAs have been shown to play an important role in the acquisition and maintenance of male fertility. For instance, deletion of Dicer in mice Sertoli cells or male germ cells leads to infertility owing to impaired spermatogenesis, progressive testicular degeneration and both meiotic and spermiogenic defects [36]–[39]. In addition, more than 200 miRNAs have been identified in the human epididymis [40]. Since the androgen level in the newborn is lower than that in the adult epididymis, the negative correlation observed between the decreasing number of miRNAs and the increasing number of mRNAs expressed in the epididymis during an individual's life time suggest that epididymal miRNAs regulate epididymal gene expression in an androgen-dependent manner [40]. Furthermore, a mammalian miRNA expression array of 26 distinct organ systems and cell types, showed that miRNAs such as mir-205, and miRNAs belonging to the miR-888 and miR-371 clusters, were significantly enriched in human reproductive systems [41]. Interestingly, the miR-888 cluster appears to play a prominent role in regulating physiological functions of the human epididymis, owing to its high degree of conservation among primates and absence from other mammalian species, and its predominant expression in the epididymis [42]. Taken together, these findings suggest a significant role of miRNAs in the regulation of epididymal functions.

Because of its high degree of region-specific gene expression, the epididymis represents an excellent model to study the influence of regulatory factors, such as miRNAs, on gene expression. The present study compared the miRNA signature found in different segments of the human epididymis with expression levels of putative mRNA targets, as determined by *in silico* analysis and by experimental confirmation. We have identified several miRNAs whose expression was either positively or negatively correlated with target gene expression pattern found in the epididymis. Our data support the hypothesis that differential expression of miRNAs along the epididymis may contribute to segment specificity of gene expression in this organ.

## Materials and Methods

### Biological Material

Human epididymides were obtained through our local organ transplantation program (Québec Tansplant, QC, Canada) following written consents of the families. The experiment are conform to the Declaration of Helsinki and were conducted according to the policies for the Human Studies with the approval of the ethical committee of the Institutional Review Board of the Centre Hospitalier Universitaire de Québec (CHUQ)(protocol 09.04.006). Three donors of 26–50 years of age with no known pathologies that could affect reproductive function were used for this study and samples were processed as previously described [4]. Briefly, artificial circulation was maintained during the surgery to preserve organ's integrity. Human epididymides were collected and processed within three hours after surgery. Tissues were dissected into three anatomical regions (caput, corpus and cauda), immediately frozen in liquid nitrogen and stored at −80°C until use. Total RNAs were extracted as previously described [4] with Trizol reagent (Invitrogen, Burlington, ON, Canada). Total RNA was purified using RNeasy mini kit columns (Qiagen, Mississauga, ON, Canada). The quality of these samples was assessed by the Agilent 2100 Bioanalyzer (Agilent Technologies, Inc., Santa Clara, CA, USA) before miRNA analysis. miRNA profiling of the caput, corpus and cauda epididymidis of three human organs was achieved with microarray technology on the total RNA extracts [4].

### miRNA microarrays

miRNA expression profiles of human epididymal tissues were generated by the Affimetrix microarray platform at the CHUL (CHUQ) Research Center (Québec, Canada). All procedures were carried out according to the manufacturer's protocol. Briefly, 1 µg of each total RNA sample was labeled with the FlashTag Biotin HSR (Genisphere, Hatfield, PA, USA) according to the manufacturer's recommendations and hybridized on the GeneChip miRNA Array (Affimetrix, Santa Clara, CA, USA). This array contains 46,228 probes comprising 7,815 probe sets and controls, and covers 71 species, including humans. GeneChip miRNA Array probes were derived from the Sanger miRBase miRNA database v11 (April 15, 2008, http://microrna.sanger.ac.uk) and include 847 human miRNAs. Hybridization was carried out according to the protocol described in the FlashTag Biotin HSR system (Genisphere). Chips were scanned with a GeneChip scanner 3000 G7 (Affymetrix) and the image data were analyzed by using the miRNA QC Tool software for quality control (www.affymetrix.com) and CEL files were imported and analyzed with the Partek Genomics Suite 6.5 software (Partek Incorporated, St Louis, MO, USA). Samples isolated from the same anatomical regions of three different epididymides were grouped and compared to each other by analysis of variance followed by a false discovery rate (FDR) correction. Microarray expression data were subjected to the following three threshold criteria: (1) Minimum Intensity - miRNAs were considered expressed if their normalized log2 intensity was ≥3.2 in at least one epididymal region; (2) Statistical Significance – miRNA expression changes were identified using a *P*-value threshold of 0.01; and (3) Differential Expression - a minimum 2-fold difference in either direction was required.

### End point and real-time PCR on selected miRNAs

Selected miRNAs showing significant variation of expression on microarrays in the different human epididymal regions were confirmed by real-time PCR with the method described by Varkonyi-Gasic and Hellens [43], [44]. This method combines the advantages of using stem-loop reverse transcription primers specific to each miRNA analyzed and a pulsed reverse transcription (RT) reaction, two parameters that increase the specificity and sensitivity of detection [45], [46]. During the first step of reverse transcription, a stem-loop RT primer is hybridized to the miRNA molecule and then reverse transcribed in a pulsed RT reaction. Briefly, 0.3 µg of RNA templates (extracted from human caput, corpus, cauda epididymidis) were denatured and mixed with 62.5 µM of each dNTP and 50 nM of the stem-loop primer at 65°C for 5 min, and then incubated on ice. First-Strand buffer, 10 mM DTT, 4 units of Protector RNase Inhibitor (Roche Diagnostics, Laval, QC, Canada) and 50 units of SuperScript II RT (Invitrogen) were added to the mixture for a total reaction volume of 20 µl. Reactions were placed on a MJ Mini thermal cycler (Bio-Rad, Mississauga, ON, Canada) and incubated for 30 min at 16°C, followed by pulsed RT of 60 cycles at 30°C for 30 s, 42°C for 30 s and 50°C for 1 s. Reverse transcriptase was then inactivated for 5 min at 85°C. During the second step, the RT product was amplified with a forward primer specific to the selected miRNA, and a universal reverse primer complementary to a section of the primer used for reverse transcription.

For end-point PCR, 1 µl of RT template was mixed with 1× ThermoPol Reaction Buffer (NEB, Pickering, ON), 50 nM of each dNTP, 0.2 µM of each primer and 2 units of Taq DNA polymerase (NEB). The reaction mixture was incubated for 2 min at 94°C, followed by 35 cycles of 15 sec at 94°C and 1 min at 60°C. A no-template control was included as a negative control. PCR products were separated on a 4% agarose gel and sequenced to control the specificity of the product according to its size and its sequence.

Real-time PCR was performed by using the SYBR Green assay on a Light Cycler (Roche Diagnostics) according to manufacturer's instructions. Briefly, SYBR Green I Master Mix was mixed with 0.5 µM of each primer and 2 µl of the RT template for a total volume of 20 µl, and placed in capillary tubes. Samples were incubated at 95°C for 5 min, followed by 40 cycles of 95°C for 5 s and 60°C for 10 s. Samples were denatured at 95°C for melting curve analysis. Fluorescence signals were acquired at 530 nm continuously from 65°C to 95°C at 0.2°C per second. A no-template control was included as negative control. Duplicate reactions were performed three times. Four points were used to plot the standard curve (non-diluted sample from the cauda epididymidis, and dilutions 1/2, 1/4 and 1/8 of the template). Data were normalized to hsa-Let-7b miRNA which is consistently and highly expressed in the different segments of the epididymis. Primers used in this study are listed in Table 1.

**Table 1:** List of primers used for the detection of miRNAs by end-point and real-time PCR and designed on the basis of the sequences of mature miRNAs (miRBase).

Mature miRNA sequences according to miRBase (http://www.mirbase.org/)	
Hsa-miR-890	UACUUGGAAAGGCAU **CAGUUG**
Hsa-miR-891a	UGCAACGAACCUGAGC **CACUGA**
Hsa-miR-891b	UGCAACUUACCUGAGU **CAUUGA**
Hsa-miR-892a	CACUGUGUCCUUUCU **GCGUAG**
Hsa-miR-892b	CACUGGCUCCUUUCUG **GGUAGA**
Hsa-let-7b	UGAGGUAGUAGGUUGU **GUGGUU**

Primers for RT	Sequences
Hsa-miR-890-RT	GTTGGCTCTG*GTGCAGGGTCCGAGGT*ATTCGCACCAGAGCCAAC**CAACTG**
Hsa-miR-891a-RT	GTTGGCTCTG*GTGCAGGGTCCGAGGT*ATTCGCACCAGAGCCAAC**TCAGTG**
Hsa-miR-891b-RT	GTTGGCTCTG*GTGCAGGGTCCGAGGT*ATTCGCACCAGAGCCAAC**TCAATG**
Hsa-miR-892a-RT	GTTGGCTCTG*GTGCAGGGTCCGAGGT*ATTCGCACCAGAGCCAAC**CTACGC**
Hsa-miR-892b-RT	GTTGGCTCTG*GTGCAGGGTCCGAGGT*ATTCGCACCAGAGCCAAC**TCTACC**
Hsa-let-7b-RT	GTTGGCTCTG*GTGCAGGGTCCGAGGT*ATTCGCACCAGAGCCAAC**AACCAC**

Primers for PCR	Sequences
Hsa-miR-890-FP	GCGGCGGTACTTGGAAAGGCAT
Hsa-miR-891a-FP	GCGGCGGTGCAACGAACCTGAGC
Hsa-miR-891b-FP	GCGGCGGTGCAACTTACCTGAGT
Hsa-miR-892a-FP	GCGGCGGCACTGTGTCCTTTCT
Hsa-miR-892b-FP	GCGGCGGCACTGGCTCCTTTCTG
Hsa-let-7b-FP	GCGGCGGTGAGGTAGTAGGTTGT
Universal primer-RP	*GTGCAGGGTCCGAGGT*

Sequences are orientated 5′ to 3′. Nucleotides in bold represent the sequences of primers used for the reverse transcription that are complementary to mature miRNAs. Underlined nucleotides represent the sequences of primers used for the PCR that are complementary to mature miRNAs. Nucleotides in italics represent the sequences complementary to universal primer.

### mRNA target prediction by *in silico* analysis

The identification of mRNA epididymal targets and correlation analyses between epididymal mRNAs and miRNAs were performed by combining the transcriptomic data obtained by microarray analysis in the caput, corpus and cauda epididymidis (published in Thimon et al, 2007) with our miRNA microarray data obtained with the exactly same samples (i.e total RNA extracts from the caput, corpus and cauda epididymidis). To understand the function of epididymal miRNAs better, putative mRNA targets of differentially expressed miRNAs were predicted from two algorithms: TargetScan (http://www.targetscan.org/) and MiRanda (http://www.microrna.org/). Only targets that were predicted by the use of both algorithms were included. Correlation analysis between miRNAs and their putative target mRNAs was carried out with the Partek Genomics Suite 6.5 software. Both Pearson and Spearman coefficients were calculated to assess the correlation between the expression of a miRNA and each of its target genes.

### Cldn10 and Esrrg 3′-UTR luciferase reporter constructs

miRNAs most frequently bind to regions located on mRNA's 3′UTRs either to prevent mRNA translation or to induce mRNA degradation. Therefore, in order to confirm the involvement of (1) hsa-miR-145 on the expression of its predicted target mRNA Cldn10 and of (2) hsa-miR-892b on Esrrg mRNA, we cloned the 3′UTR regions of these targets in a luciferase reporter system. The rationale for using this assay is that the binding of a given miRNA to its specific mRNA target site will repress luciferase protein production thereby reducing its activity or expression that can be measured and compared to a control. Briefly, the 3′UTR region of Cldn10 was cloned and inserted in a multi-cloning site downstream of a luciferase translation sequence into the pMIR-REPORT™ miRNA Expression Reporter Vector System (Applied Biosystem, Life Technologies Corporation, Carlsbad, CA, USA). Human testicular genomic DNA was used along with Taq Phusion polymerase (New England Biolabs, Pickering, ON, Canada) to obtain the desired sequence. The primers used for the amplification of the Cldn10 3′-UTR region included SpeI and HindIII restriction enzyme recognition sites and corresponded to: F-Cldn10-SpeI 5′ GGACTAGTCCAAGAGCTCGCTGGCAAGCTG 3′ and R-Cldn10-HindIII 5′ CCCAAGCTTAGGTATGGAATTTTTTTTAT 3′. Competent DH5-α E. coli bacteria were transformed with the recombinant plasmid by heat shock and screened on ampicillin-containing medium. Plasmids positive for the 1.7 kb insert were sequenced to confirm expression and accuracy of the Cldn10 3′-UTR. A similar procedure was followed for the construction and isolation of the p-Mir-report Esrrg 3′-UTR expression vector. The primers used to amplify the Esrrg 3′-UTR were F-Esrrg-SpeI 5′ GGACTAGTCCTATAATAATCCAGGAGTTGC 3′ and R-Esrrg-HindIII 5′CCCAAGCTTCATAATAATTTGAGTTACAC 3′. Resulting constructions were named P-miR-Cldn10 and P-miR-Esrrg respectively.

### Cell culture and transfection

Immortalized human epididymal epithelial cells [47] were used for experimental confirmation of the role of miR-145 on the expression of Cldn10 and miR-892b on the expression of Esrrg. Functional analysis was performed on the FHCE1 cell line originating from the caput epididymidis of fertile donors and expressing epididymal markers [47]. Cells at passage 9–11 were seeded on 24-well plates and maintained at 32°C and 5% (v/v) CO_2_ in Dulbecco's minimum essential medium (DMEM; Invitrogen) containing antibiotics and supplemented with 10% (v/v) Fetal Bovine Serum and other components detailed in [47]. 24 hours later, co-transfection of 0.4 ug of plasmid DNA clones (control plasmid without miRNA target sequences, P-miR-Cldn10 or P-miR-Esrrg) with synthetic miRNAs that mimic endogenous miRNAs (5 nM Syn-hsa-miR-145 miScript miRNA Mimic or 5 nM Syn-hsa-miR-892b miScript miRNA Mimic (Qiagen, Toronto, ON, Canada)) was performed by using the Attractene transfection reagent (Qiagen) according to the manufacturer's protocol. Transfections of plasmid DNAs without miRNA Mimic, or co-transfection of a miRNA Mimic with a plasmid that did not contain its corresponding binding site, were used as controls. Following incubation with transfection reagents for 5 hours, the medium was replaced and 43 hours later, lysates of FHCE1 cultures from all treatment groups (four replicates per condition) were collected. Firefly luciferase activity was analyzed by the luciferase assay system (Luciferin D-Firefly, U.S. Biological, Swampscott, MA, USA) and luminescence was measured in a Luminoskan Ascent luminometer (Thermo Scientific, Nepean, ON, Canada). Three independent experiments were performed for each miRNA/target mRNA matching set. Data were compared by one way ANOVA followed by a Newman-Keuls multiple comparison test by using Prism software, Version 4.0. (Prism software corporation, Irvine, CA, USA).

## Results

### Pattern of miRNA expression along the epididymis

**Figure 1:**
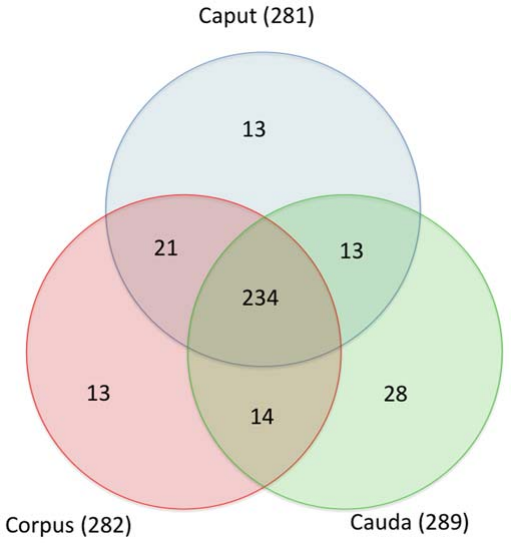
Venn diagram comparing miRNA expression in three human epididymal regions (caput, corpus and cauda). A threshold has been applied to detect miRNAs expressed with a Log 2 intensity >3.2 in at least one epididymal region.

**Figure 2:**
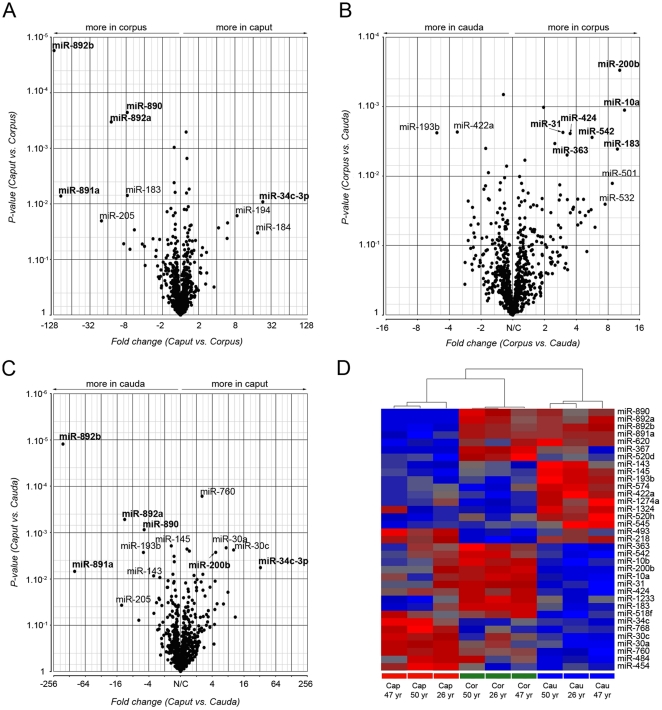
miRNA profiling in discrete segments of the epididymis reveals important differences regarding miRNA spatial distribution. (A), (B) and (C): Volcano plots displaying miRNAs with large-magnitude changes (x-axis) as well as high statistical significance (y-axis) between the caput and corpus, corpus and cauda, and caput and cauda epididymidis, respectively. (D): Hierarchical clustering of the 35 miRNAs that best define the different epididymal regions (Fold change >2, *P*<0.01). Each cell in the matrix represents the expression level of a single miRNA in a single sample from each donor, with red and blue indicating intensity level above and below the median for this miRNA across all samples, respectively. Cap: caput, Cor: corpus, Cau: cauda. 26 yr, 47 yr and 50 yr: donors of 26, 47 and 50 years old.

Using a global miRNA microarray approach we determined the miRNA signature found in the human caput, corpus and cauda epididymides. Three individuals were included in this study. Among the 847 miRNAs spotted on the chip, 336 were detected in the epididymis (Table S1); 281, 282 and 289 miRNAs in the caput, corpus and cauda region, respectively (Fig. 1). Most of these miRNAs were detected in all regions of the epididymis, while 17% (54/336) were detected in only one of the three regions (Fig. 1, Table S1). The miRNA profiles of the different epididymal regions showed strong variations from one region to another (Fig. 2). Some miRNAs displayed a pronounced change of expression, as illustrated by marked changes and low p-values on the volcano plots (Fig. 2 A, B and C). For instance, miR-892b and miR-891a were highly and significantly (p<0.01) more expressed in the cauda than in the caput epididymidis, with 170- and 102-fold changes, respectively (Fig. 2 A and C). In contrast, the expression of miR-34c-3p was 97- and 102-fold greater in the caput epididymidis than in the corpus and cauda regions, respectively (Fig. 2 A and C). In addition, changes were observed between the corpus and cauda regions with miR-200b, miR-10a, miR-424, miR-542, miR-31, miR-183 and miR-363 being significantly over-expressed in the corpus versus the cauda region (Fig. 2 B). miRNAs with a differential expression of at least two with a p-value below 0.01 were clustered in relation to their intensity profiles (Fig. 2 D). Overall, 35 miRNAs displayed significant changes in expression from one epididymal region to another, with higher levels of 17 miRNAs in the caput/corpus regions and 18 in the cauda. Dendrogram classification grouped samples according to similar miRNA expression profiles (Fig. 2 D). Three groups of samples were distinguished and low inter-individual variations were observed among the three donors (Fig. 2 D).

### Members of the miR-888 cluster and miR-215 are differentially expressed in the epididymis

**Figure 3:**
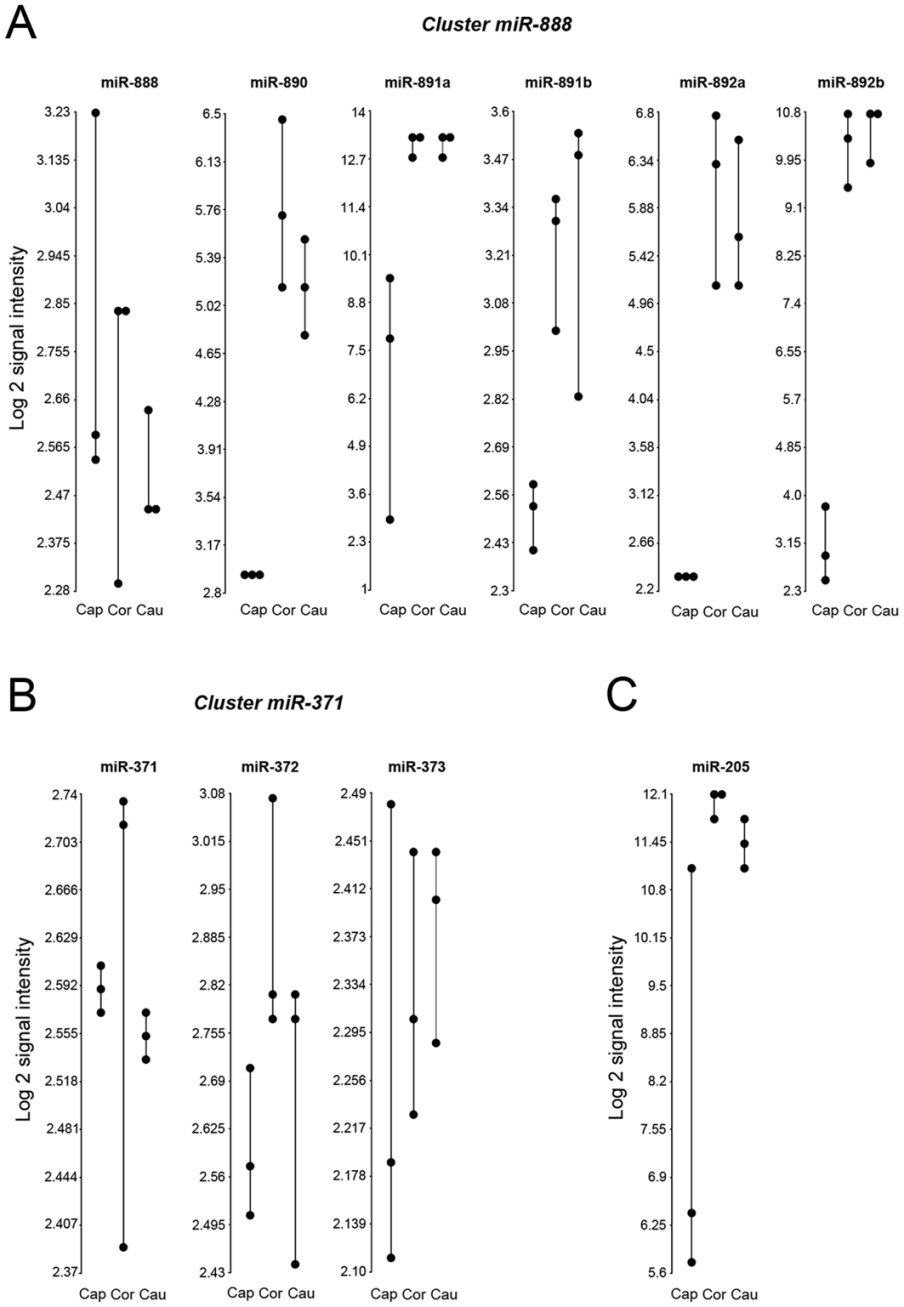
mir-205 and members of the miR-888 cluster are differentially expressed in the epididymal tubule. (A) (B) and (C): Dot plots showing the Log 2 signal intensity of miRNAs belonging to the miR-888 and miR-371 cluster families, and miR-205 in the different epididymal regions: caput (cap), corpus (cor) and cauda (cau). Each dot represents the intensity of a selected miRNA in one sample. Different samples providing from the same anatomical regions of the 3 donors are joined by straight lines.

On the basis of small RNA libraries sequenced from 26 distinct organ systems, several miRNAs, including miR-205 and members of the miR-888 and miR-371 clusters, are considered to be expressed primarily in reproductive tissues [41]. Our results indicate that members of the miR-888 cluster (i.e. miR-890/miR-891a/miR-891b/miR-892a/miR-892b) and miR-205 exhibit significantly lower levels in the caput relative to the corpus, while the levels of the miR-371 cluster did not differ (Fig. 3 A, B and C). Five of six miRNAs located in the miR-888 cluster displayed changes ranging from 1.7-fold for miR-891b to 126-fold for miR-892b, between the caput and the corpus region (miR-890/miR-891a/miR-891b/miR-892a/miR-892b). However, miR-888 itself did not show any significant variation from one region to another (Fig. 3 A, Fig. S1). miR-205 followed the same pattern of expression as miR-890/miR-891a/miR-891b/miR-892a/miR-892b, with higher levels in the distal regions of the epididymis (Fig. 3 C). No significant changes were observed for miR-371, miR-372 and miR-373, which are part of the miR-371 cluster (Fig. 3 B).

Overall, these results show that the levels of miR-890/miR-891a/miR-891b/miR-892a/miR-892b and miR-205, previously reported to be primarily expressed in reproductive tissues, are present at high levels in the distal region of the epididymis, suggesting a role of these microRNAs in the later stages of epididymal sperm maturation or storage.

### Confirmation of the microarray data by end-point and quantitative PCR for the miR-888 cluster

**Figure 4:**
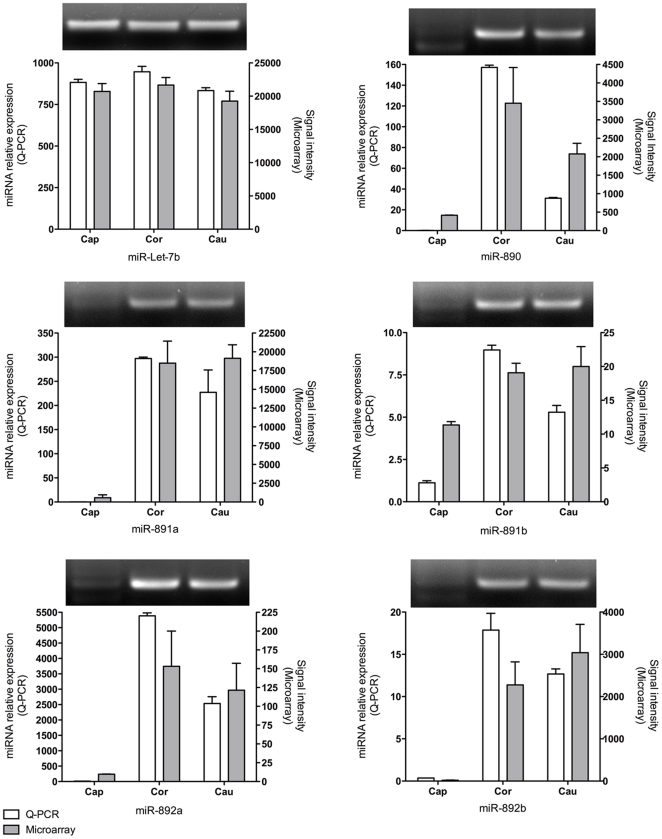
Expression of the miR-888 cluster family confirmed by end-point and real-time PCR. The expression of miR-890, miR-891a, miR-891b, miR-892a and miR-892b was assessed in total RNA extracts from the human caput, corpus and cauda epididymis from three donors. Results obtained by real-time PCR (Q-PCR) were compared with the data obtained in the microarray study (microarray). Real-time PCR data were normalized to the expression of miR-Let-7b, which is highly and consistently expressed along the epididymis. For each miRNA, end-point PCR products after 35 cycles are shown.

Expression of members of the miR-888 cluster was confirmed by end-point and quantitative PCR (Fig. 4). To this aim, a method optimized for the detection of small RNAs by PCR and adapted from Varkonyi-Gasic and Hellens (2010) was used [44]. miR-Let-7b, which is consistently and highly expressed all along the epididymis was used as a control to normalize real time PCR data (Fig. 4).

Both end-point and relative quantification by real time PCR of miR-890/miR-891a/miR-891b/miR-892a/miR-892b confirmed the microarray data (Fig. 4). Very low signals for miR-890/miR-891a/miR-891b/miR-892a/miR-892b were observed by PCR in the caput, whereas strong signals were detected in the corpus and cauda. Although the levels between the two methods of detection used (Real time PCR vs microarray) were different, the trend of expression for each miRNA was the same.

### mRNAs targets of epididymal miRNAs

**Figure 5:**
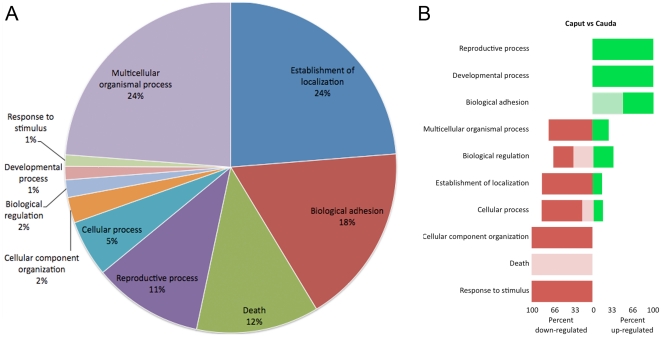
Categorization of predicted mRNA targets according to their GO terms. (A) Pie chart grouping predicted mRNA targets of miRNAs that significantly vary from one epididymal region to another (Fold change >2, p value<0.01). (B) Forest plot showing the categories of predicted mRNA targets that are significantly up or down regulated between the caput and cauda epididymidis (P value≤0.001). Light colors: miRNAs populations with a fold change ranging from 2 to 4. Dark colors: miRNAs populations with a fold change above 4.

*In silico* prediction of target mRNAs was performed by using algorithms based on sequence complementarity between miRNAs and miRNA-binding sites. Target mRNAs were indicated from our previous transcriptomic analysis performed on human epididymal samples from the caput, corpus and cauda regions [4]. Classification of epididymal mRNAs predicted to be regulated by miRNAs indicated that most mRNA targets belonged to the categories of “Multicellular organismal process”(24%), “Establishment of localization” (24%), “Biological adhesion” (18%), “Death” (12%), and “Reproductive process” (11%) (Fig. 5A). All of the target genes belonging to the “Reproductive process” category were up-regulated in the caput relative to the cauda epididymidis (Fig. 5 B). In addition, 100% of the target genes belonging to the “Death” category were down-regulated in the caput relative to the cauda region (Fig. 5 B). Of predicted mRNAs target genes, several exhibited a segment-specific expression in the epididymis (Fig. S2). For instance, the serine/threonine protein kinase homeodomain-interacting protein kinase 2 (Hipk2)(more expressed in the caput epididymidis), Lysophosphatidic acid receptor 5 (Lpar5) (more expressed in the corpus epididymidis), and Gap junction gamma-1 protein (Gjc1) (more expressed in the cauda epididymidis), were predicted to be targeted by epididymal miRNAs.

### Correlation between the expression of epididymal miRNAs and their predicted targets

**Figure 6:**
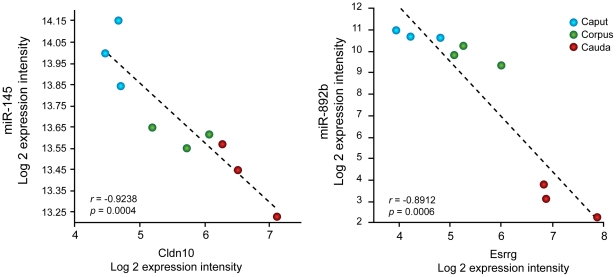
Correlation between the expression of Cldn10 and Esrrg with the expression of miR-145 and miR-892b. Correlation was based on the mRNA and miRNA expression intensities detected in three epididymal segments (Caput, corpus or cauda) from three donors. Each dot represents the Log 2 intensity from one segment of one donor.

Human miRNAs repress gene expression by pairing with complementary sequences located within the 3′ untranslated regions (3′ UTR) of targeted mRNAs, triggering either a translational repression and mRNA degradation [48] or, to a lower extent, a translational activation depending on the cell's physiological conditions [34], [35]. Of miRNAs expressed in the human epididymis, we focused our interest on miRNAs whose intensity was significantly different from one region to another (fold change ≥2, *P*≤0.01) and whose mRNA targets were differentially expressed in this organ (fold change ≥2, *P*≤0.001). We identified 25 miRNAs/target mRNA pairs whose expressions were positively (9) or negatively (16) correlated (p-value≤0.05; Tables 2 and 3, Fig. S3 and S4). Of these target genes, 60% belonged to the category of membrane proteins including receptors, transporters, channels and transmembrane proteins. In addition, several genes were predicted to be regulated by the miR-888 cluster family. For instance, Major CDK9 elongation factor-associated protein (Aff4) and Zinc finger E-box-binding homeobox 1 (Zeb1) were positively correlated with miR-891a and miR-892a respectively, while Estrogen-related receptor gamma (Esrrg), Sperm associated antigen 8 (Spag8), Glycine receptor subunit beta (Glrb), Cysteine-rich secretory protein LCCL domain-containing 1 (Crispld1), Transmembrane protein 68 (Tmem68), and Zinc finger protein 395 (Znf395), were negatively correlated with different members of the miR-888 cluster (Tables 2 and 3, Fig. S3 and S4). The gene encoding for Claudin10 (Cldn10) is predicted to be regulated by miR-145 whose expression is negatively correlated with this gene (Fig. 6). In addition, the gene encoding for estrogen-related receptor gamma (Esrrg) was negatively correlated with two members of the miR-888 cluster, miR-892b (Tables 2 and 3, Fig. 6, Fig. S3) and miR-891b (Tables 2 and 3, Fig. S3). Interestingly, CLDN10 is a protein of interest regarding epididymal functions and fertility since it is localized in tight junctions in the human epididymis and might be involved in the formation of the blood-epididymal barrier [1]. Considering the role of Cldn10 in epididymal functions and the presumed importance of cluster miR-888 target genes such as Esrrg, we focused our interest on these two genes and conducted an experimental approach to determine if Cldn10 was regulated by miR-145 and Esrrg by miR-892b.

**Table 2:** List of selected miRNAs that displayed a positive correlation with their predicted target mRNAs.

miRNAs	Target genes	Description	*Pearson correlation's coefficient*	*P-value*
Hsa-miR-768	Tsga10	Testis-specific gene 10 protein	0.9558	<0.0001
Hsa-miR-193	Atrnl1	Attractin-like protein 1	0.9511	<0.0001
Hsa-miR-145	Ptger3	Prostaglandin E2 receptor EP3 subtype	0.8802	0.0009
Hsa-miR-143	Slc16a1	Monocarboxylate transporter 1	0.8324	0.0027
Hsa-miR-891a	Aff4	Major CDK9 elongation factor-associated protein	0.7575	0.0091
Hsa-miR-520h	Tmem200b	Transmembrane protein 200B	0.7312	0.0126
Hsa-miR-620	Cldn1	Senescence-associated epithelial membrane protein	0.6439	0.0306
Hsa-miR-545	Pde6d	Retinal rod rhodopsin-sensitive cGMP 3′,5′-cyclic phosphodiesterase subunit delta	0.6362	0.0327
Hsa-miR-892a	Zeb1	Zinc finger E-box-binding homeobox 1	0.5747	0.0454

**Table 3:** List of selected miRNAs that displayed a negative correlation with their predicted target mRNAs.

miRNAs	Target genes	Description	*Pearson correlation's coefficient*	*P-value*
Hsa-miR-145	Cldn10	Claudin-10	−0.9238	0.0004
Hsa-miR-200	Gjc1	Gap junction gamma-1 protein	−0.9207	0.0002
Hsa-miR-484	Mrc1	Macrophage mannose receptor 1	−0.9191	0.0002
Hsa-miR-892b	Esrrg	Estrogen-related receptor gamma	−0.8912	0.0006
Hsa-miR-30c	Tes	Testin	−0.8812	0.0008
Hsa-miR-30a	Rab23	Ras-related protein Rab-23	−0.8709	0.0011
Hsa-miR-891b	Esrrg	Estrogen-related receptor gamma	−0.7542	0.0094
Hsa-miR-424	Tmem181	Transmembrane protein 181	−0.7536	0.0095
Hsa-miR-892a	Spag8	Sperm associated antigen 8	−0.7996	0.0048
Hsa-miR-183	Cacna1c	Voltage-dependent L-type calcium channel subunit alpha-1C	−0.7025	0.0174
Hsa-miR-892a	Glrb	Glycine receptor subunit beta	−0.7018	0.0175
Hsa-miR-145	Cftr	Cystic fibrosis transmembrane conductance regulator	−0.6833	0.0425
Hsa-miR-890	Crispld1	Cysteine-rich secretory protein LCCL domain-containing 1	−0.6459	0.0301
Hsa-miR-892b	Tmem68	Transmembrane protein 68	−0.6211	0.0371
Hsa-miR-890	Znf395	Zinc finger protein 395	−0.6095	0.0407
Hsa-miR-760	Glipr1	Glioma pathogenesis-related protein 1	−0.5749	0.0427

### Experimental confirmation of target genes regulation by miR-145 and miR-892b using a luciferase reporter system

**Figure 7:**
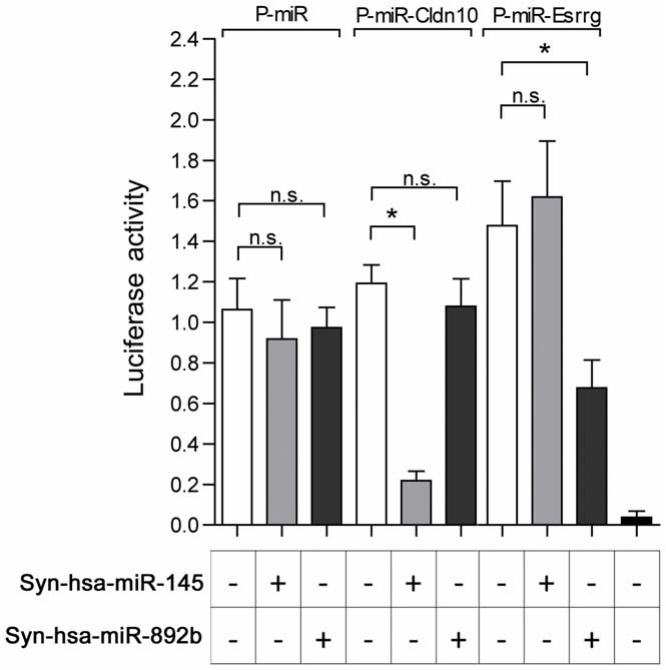
Luciferase Reporter assay performed on FHCE1 immortalized human epididymal cell line. A control plasmid (P-miR), or plasmids containing the 3′UTR sequences of Cldn10 (P-miR-Cldn10) or Esrrg (P-miR-Esrrg) were co-transfected in the presence (+) or in the absence (−) of mimics Syn-Hsa-miR-145 or Syn-Hsa-miR-892b. Values of control non transfected cells are indicated in the last column. Data are means ± SEM from three independent experiments. ns: non significant, *: P-value≤0.05.

In order to confirm the role of miR-145 on the expression of Cldn10 and miR-892b on Esrrg, we conducted a series of co-transfection experiments by using mimic Syn-hsa-miR-145 and Syn-hsa-miR-892b along with a luciferase reporter system containing the 3′UTR sequence of Cldn10 or Esrrg (Fig. 7). In this system, luciferase activity was used as a read-out assay: the binding of a synthetic mimic miRNA to its specific mRNA target site located in the 3′UTR sequence inhibits luciferase protein production and subsequently reduces its activity/expression. After co-transfection of the control plasmid (P-miR) that does not contain any 3′UTR sequence along with synthetic miRNAs, no significant loss of luciferase activity was observed compared to control condition in absence of synthetic miRNAs (Fig. 7, P-miR). Therefore, synthetic miRNAs do not modify the expression of luciferase in a non-specific manner.

Co-transfection of P-miR-Cldn10 with hsa-miR-145, caused a significant decrease in luciferase activity compared with P-miR-Cldn10 transfected in the absence of hsa-miR-145. Luciferase activity was not affected by the presence of miR-892b which does not have any binding sites on the 3′UTR of Cldn10 (Fig. 7, P-miR-Cldn10). These results suggest that hsa-miR-145 significantly and specifically affects the expression of Cldn10 at the post-transcriptional level. Co-transfection of P-miR-Esrrg with hsa-miR-892b resulted in a significant decrease in luciferase activity relative to P-miR-Esrrg transfected alone. Luciferase activity was not affected by the presence of hsa-miR-145 which does not bind the 3′UTR of Esrrg (Fig. 7, P-miR-Esrrg). These data suggest that hsa-miR-892b significantly and specifically affects the expression of Esrrg at the post-transcriptional level. We therefore confirmed the respective roles of miR-145 and miR-892b, in the post-transcriptional regulation of Cldn10 and Esrrg.

## Discussion

Sperm maturation occurs in the epididymis and requires the interaction of spermatozoa with proteins that are synthesized and secreted by the epididymal epithelium in a highly regionalized manner. The resulting microenvironments are dependent on the elevated degree of regionalized gene expression in the epididymis [1]–[4], [49], [50], which constitutes one of the most intriguing aspects of this organ. The role of androgens and lumicrine factors, including spermatozoa, on epididymal gene expression has been extensively explored. However, the contribution of RNA interference and regulation of mRNA stability in the epididymis are poorly understood. Using the microarray technology on human epididymides, we have explored the pattern of expression and the role of epididymal miRNAs. We conducted an integrated study by combining the previously published transcriptomic data from 3 epididymal regions of 3 donors [4], with the corresponding miRNA profiles found in the same samples. Since miRNAs can regulate either the translation of a target mRNA or its stability, it is likely that regulation of some target genes may occur at the translational level without affecting the mRNA level. These types of changes would therefore not be measured in microarray analyses. However, since changes in the expression of miRNAs are generally negatively or positively correlated with changes in the expression levels of their targeted mRNAs [51]–[54], we assessed the correlation between epididymal miRNAs and their mRNA targets detected in the different epididymal segments by using *in silico* miRNA/target mRNA predictions. We determined that the expression of miRNAs differs in the distinct regions of the human epididymis (i.e. caput, corpus and cauda) and is correlated with the levels of target mRNAs in the same regions. Of the 847 human miRNAs analyzed, 35 were differentially expressed in the distinct segments of the epididymis. Of these, 5 members of the miR-888 cluster (miR-890, miR-891a/b, miR-892a/b) were significantly more abundant in the corpus/cauda regions of the epididymis, suggesting a role in the regulation of the later stages of epididymal sperm maturation. Interestingly, miRNAs belonging to the miR-888 cluster have been found in primates and their expression is restricted to the epididymis [41], [42]. These miRNAs have been implicated in epididymal functions such as sperm maturation, morphogenesis and establishment of primate-specific epididymal characteristics, on the basis of *in silico* target predictions [42]. Of previously predicted mRNA targets of the miR-888 cluster, we found that Spag8 (sperm associated antigen 8) mRNA expression encoding for a human sperm membrane protein (hSMP-1) was significantly and negatively correlated with the expression of miR-892a along the human epididymis (Tables 2 and 3, Fig. S3). In contrast to miR-892a, the expression of Spag8 was significantly decreased in the corpus and cauda relative to the caput epididymidis (Fig. S3). The involvement of hSMP-1 in the testis-specific process of spermatogenesis [55] is coherent with the down-regulation of its expression by miR-892a in the epididymis but this needs to be experimentally confirmed.

Among other target genes of the miR-888 cluster, we identified Crispld1 (Cysteine-rich secretory protein LCCL domain-containing 1), Tmem68 (Transmembrane protein 68), Znf395 (Zinc finger protein 395)(Tables 2 and 3, Fig. S3), Zeb1 (Zinc finger E-box-binding homeobox 1), Aff4 (Major CDK9 elongation factor-associated protein)(Tables 2 and 3, Fig. S4) and Esrrg (Estrogen related receptor Gamma)(Tables 2 and 3, Fig. S3). Esrrg expression was negatively correlated with the expression of both miR-892b and miR-891b. This gene encodes an orphan nuclear steroid hormone receptor previously identified as estrogen-related receptor gamma, for which no endogenous ligand has been determined [56], [57]. Interestingly, targeted inactivation of Esrrg in mouse embryos triggers impaired development of the kidney [56], an organ that shares a common embryologic origin and characteristics with the epididymis. Whereas we demonstrated that Esrrg expression is regulated at the post-transcriptional level by the epididymis-specific miR-892b, the role and significance of Esrrg in the epididymis has to be investigated.

It is well accepted that miRNAs affect gene expression after pairing with sequences located on the 3′UTR region of targeted mRNAs, resulting in translational repression and subsequent mRNA degradation [25]. To date, few examples of miRNAs increasing the transcription or translation of target genes and resulting in positive correlations between miRNAs and target mRNAs have been described [33], [35], [58]. In our study, almost 40% of epididymal miRNAs exhibited a significant positive correlation with their predicted target mRNAs. However, it is not known whether the effects are direct or indirect. The fact that individual miRNAs may target multiple mRNAs and that individual mRNAs may be targeted by multiple miRNAs creates the potential for a complex network of interactions in epididymal cells with positive and negative feedback loops.

Among mRNA targets that were negatively correlated with the levels of their corresponding miRNAs, Cldn10 was particularly interesting with regards to epididymal function and fertility [1], [59]. Cldn10 has been shown to be involved in the paracellular transport of cations across epithelial tight junctions [60], [61] and is expressed in tight junctions of the human epididymis [1], [59]. Tight junctions are essential to epididymal physiology since they participate to the formation of the blood-epididymis barrier that selectively excludes or concentrates ions and molecules [62], [63]. This barrier controls the composition of the epididymal fluid and provides protection to auto-antigenic spermatozoa in an immuno-privileged environment. Interestingly, Cldn10 mRNA expression is altered in patients with obstructive azoospermia [64] and the localization of Cldn10 in the epididymal epithelium is modified in infertile patients with non-obstructive azoospermia [65]. Our observation regarding the regulation of Cldn10 by miR-145 therefore opens new avenues on the factors that can affect epididymal functions that are essential for proper sperm maturation.

Regulation of epididymal gene expression controls the physiological functions taking place in the different regions of epididymis that generate mature and fertile spermatozoon. Overall, our results provide support for a role of miRNAs in the establishment and maintenance of a distinctive gene expression pattern along the human epididymis and, therefore, their contribution to sperm maturation and acquisition of fertilizing ability. Our study sheds new light on the well-orchestrated regulation of gene expression that occurs in the epididymis and provides a broad foundation for further investigations on the molecular mechanisms that affect male fertility.

## Supporting Information

Figure S1 **Expression level of members of the miR-888 cluster family on microarrays.** Data represent expression intensities found in the different segments of the epididymis (Caput, corpus and cauda) from three donors. Data are means ± SEM. *: P-value≤0.05, **: P-value≤0.01, ***: P-value≤0.001.
(TIF)

Figure S2 **Hierarchical clustering of predicted mRNA targets that are differentially expressed along the human epididymis.** Only mRNAs with a ≥2 fold change and a P-value≤0.001 are clustered. Each cell in the matrix represents the expression level of a single mRNA in a single sample from each donor, with red and blue indicating intensity level above and below the median for this mRNA across all samples, respectively. Cap: caput, Cor: corpus, Cau: cauda. 26 yr, 47 yr and 50 yr: donors of 26, 47 and 50 years old.
(TIF)

Figure S3 **Negative correlation between selected miRNAs and their predicted mRNA targets.** Data represent the Log 2 expression found in the different segments of the epididymis (Caput, corpus and Cauda). Data are means ± SEM.
(TIF)

Figure S4 **Positive correlation between selected miRNAs and their predicted mRNA targets.** Data represent the Log 2 expression found in the different segments of the epididymis (Caput, corpus and Cauda). Data are means ± SEM.
(TIF)

Table S1 **List of miRNAs with a mean Log 2 signal intensity above the threshold of 3.2 in the different epididymal regions (Caput, corpus, and cauda) according to microarray analysis.** – indicates miRNAs with a mean Log 2 signal intensity below 3.2.
(XLS)
