# Supplementary figures and images for: Role of MicroRNAs in Controlling Gene Expression in Different Segments of the Human Epididymis
Source: PLoS One. 2012 Apr 12;7(4):e34996. doi: 10.1371/journal.pone.0034996 (PMC3325285; doi:10.1371/journal.pone.0034996)

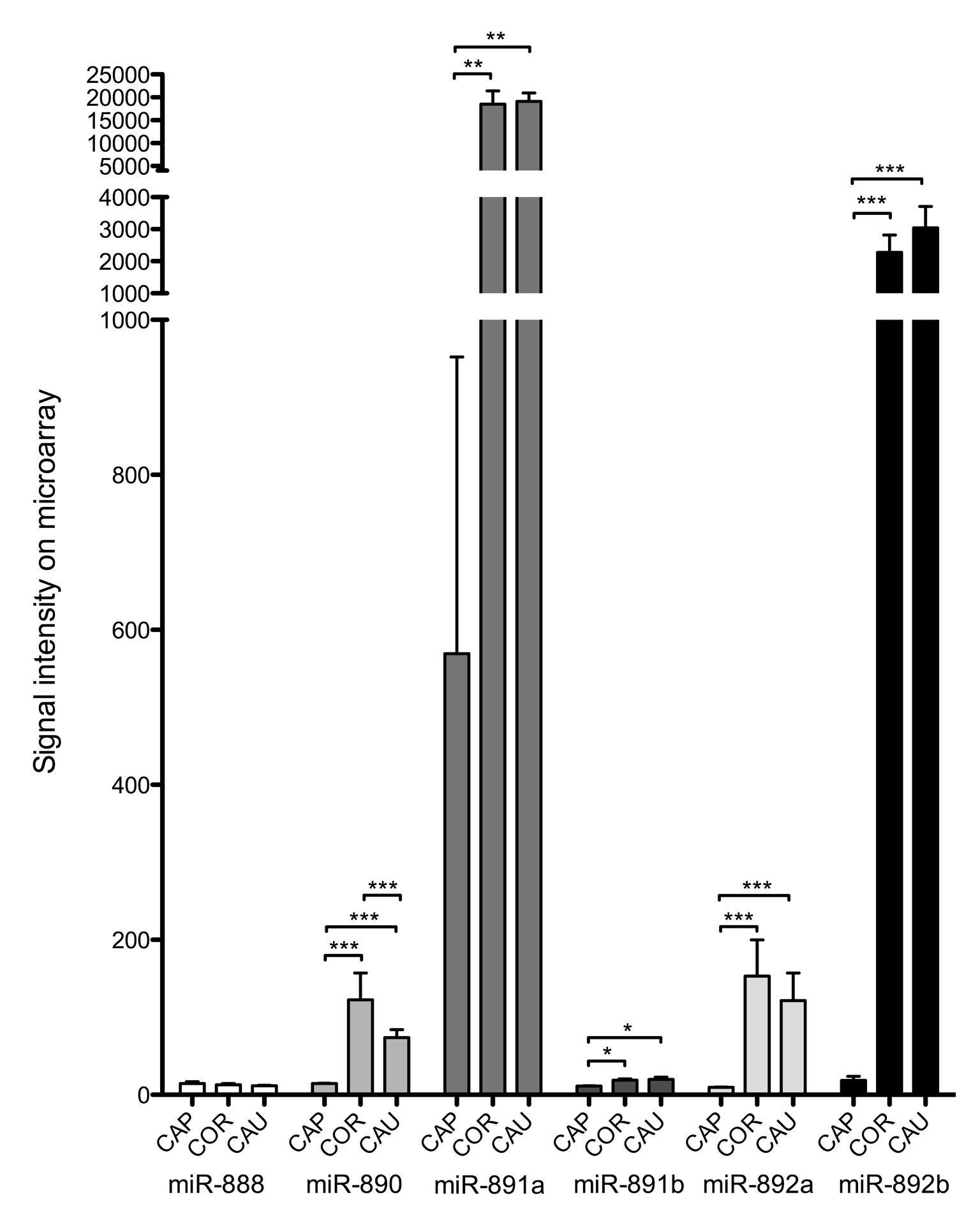

Supplement: Figure S1 — Expression level of members of the miR-888 cluster family on microarrays. Data represent expression intensities found in the different segments of the epididymis (Caput, corpus and cauda) from three donors. Data are means ± SEM. *: P-value≤0.05, **: P-value≤0.01, ***: P-value≤0.001. (TIF) [file pone.0034996.s001.tif]

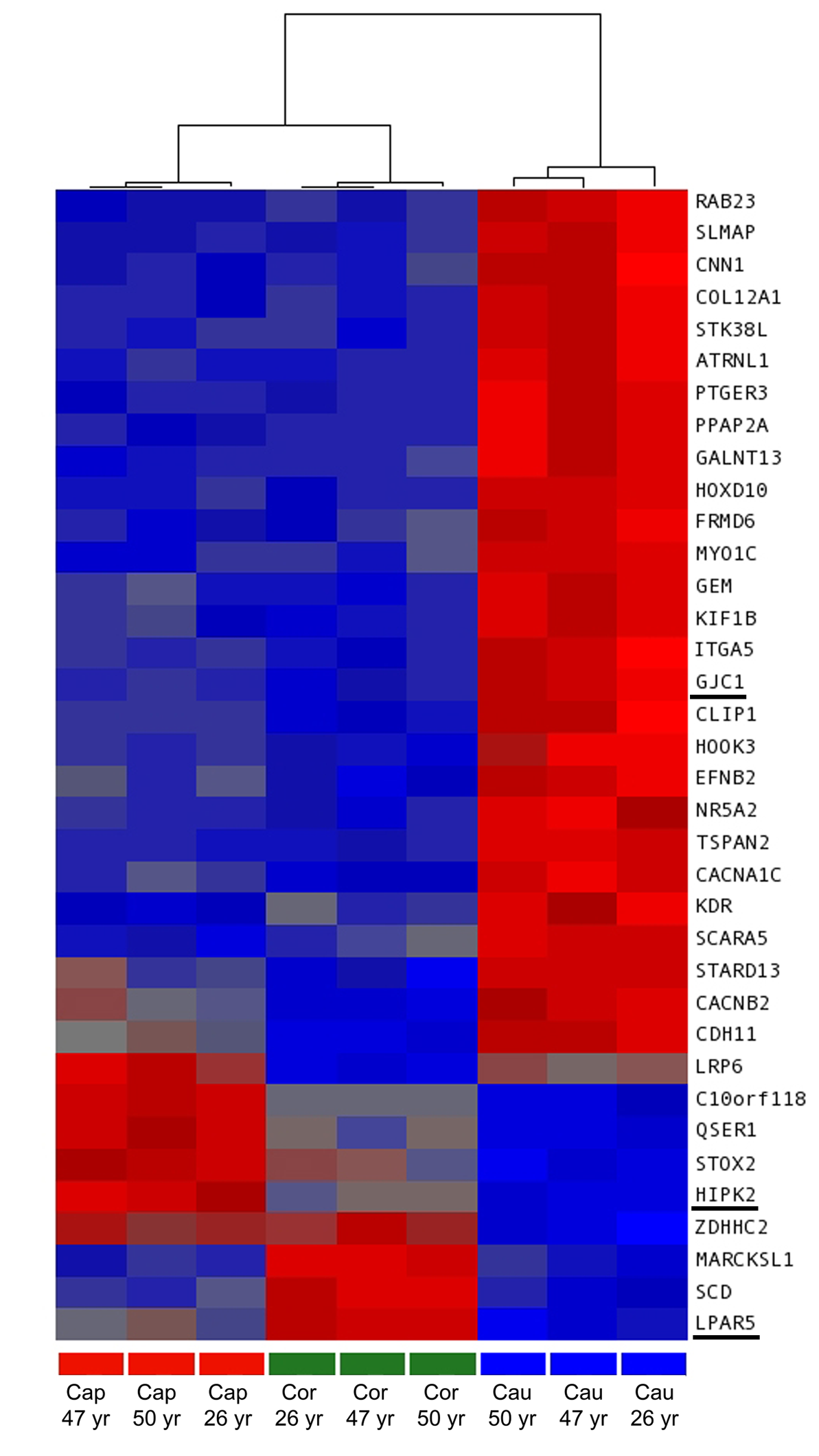

Supplement: Figure S2 — Hierarchical clustering of predicted mRNA targets that are differentially expressed along the human epididymis. Only mRNAs with a ≥2 fold change and a P-value≤0.001 are clustered. Each cell in the matrix represents the expression level of a single mRNA in a single sample from each donor, with red and blue indicating intensity level above and below the median for this mRNA across all samples, respectively. Cap: caput, Cor: corpus, Cau: cauda. 26 yr, 47 yr and 50 yr: donors of 26, 47 and 50 years old. (TIF) [file pone.0034996.s002.tif]

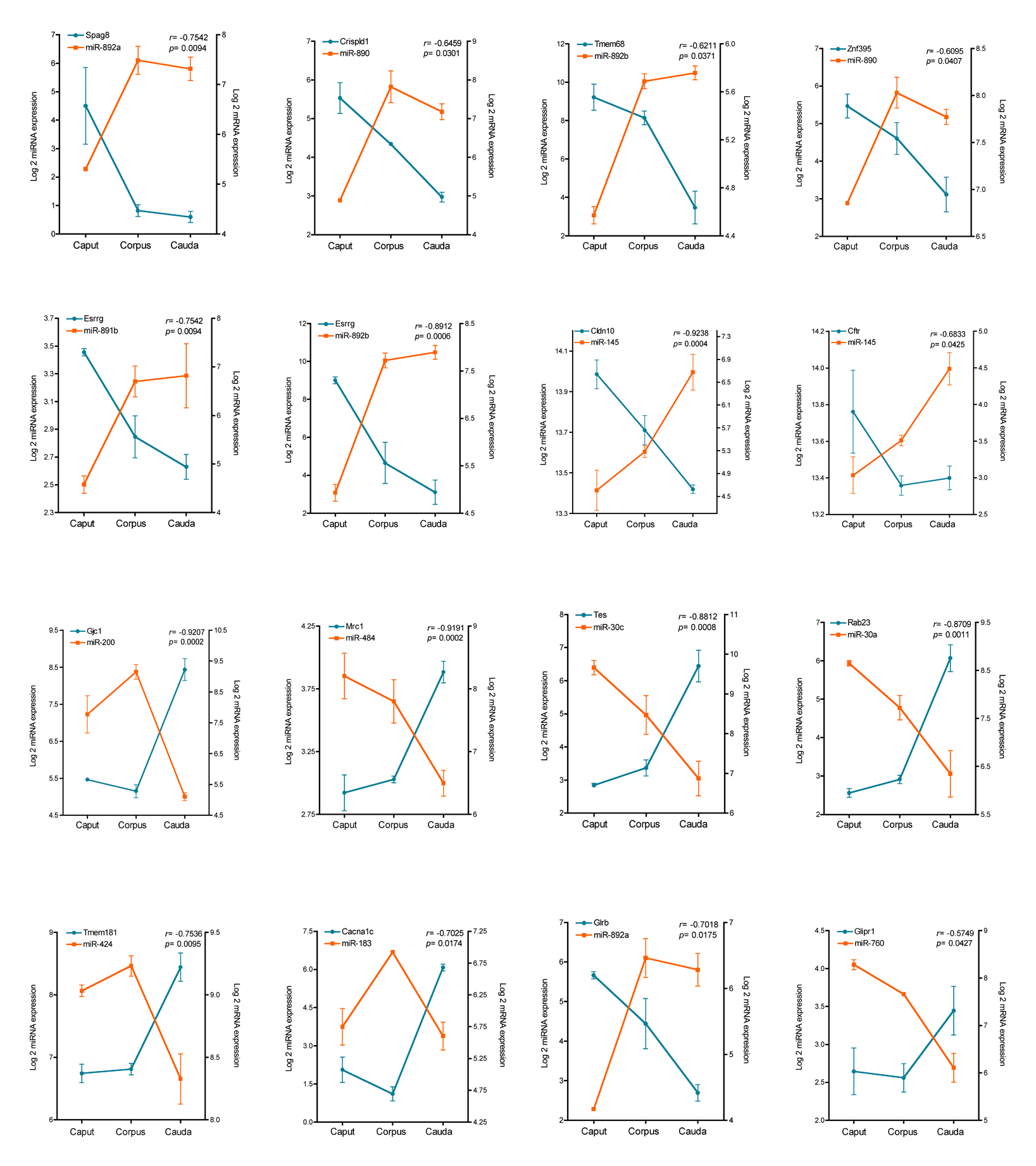

Supplement: Figure S3 — Negative correlation between selected miRNAs and their predicted mRNA targets. Data represent the Log 2 expression found in the different segments of the epididymis (Caput, corpus and Cauda). Data are means ± SEM. (TIF) [file pone.0034996.s003.tif]

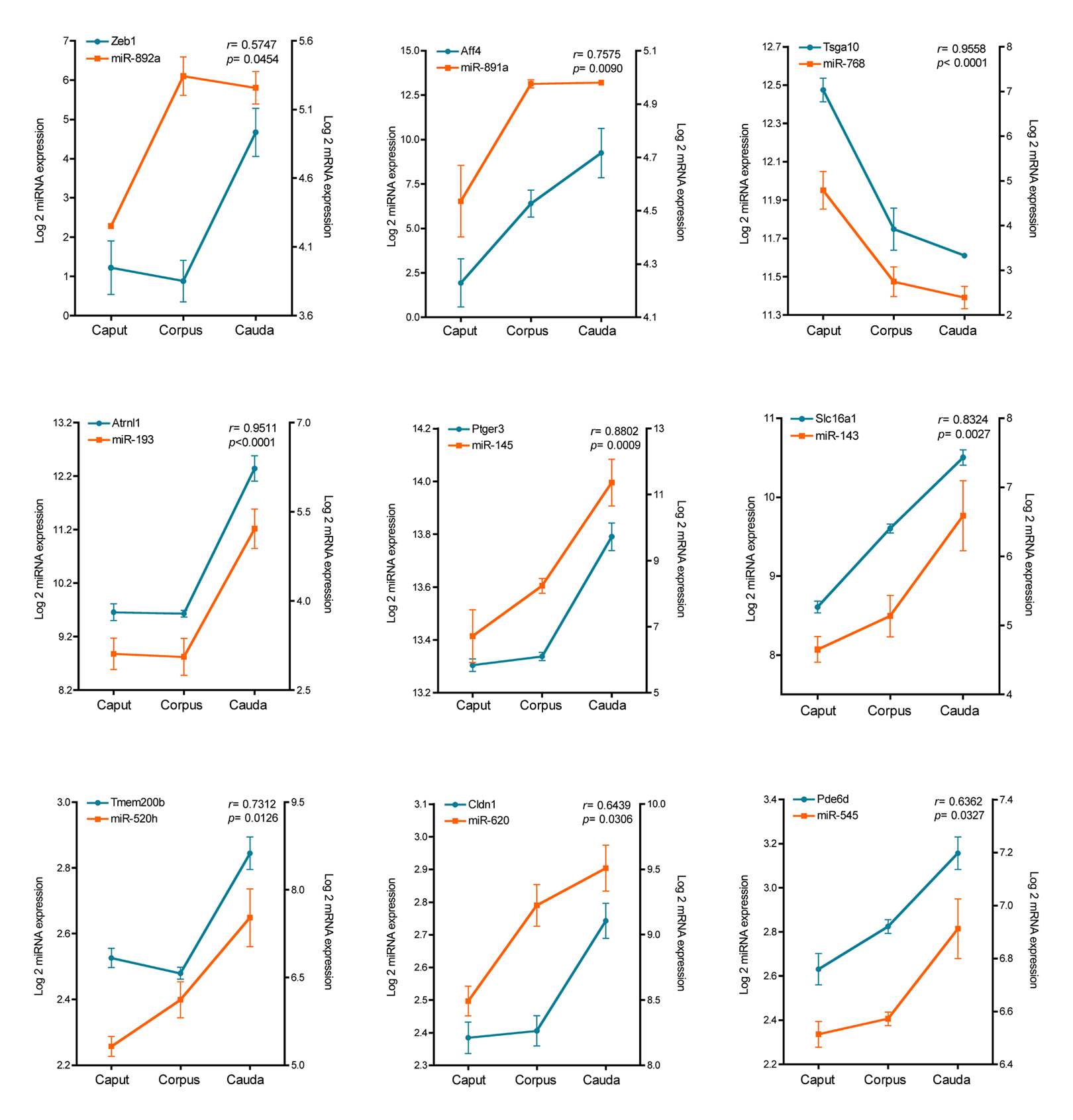

Supplement: Figure S4 — Positive correlation between selected miRNAs and their predicted mRNA targets. Data represent the Log 2 expression found in the different segments of the epididymis (Caput, corpus and Cauda). Data are means ± SEM. (TIF) [file pone.0034996.s004.tif]
